# YOLO-MARS: An Enhanced YOLOv8n for Small Object Detection in UAV Aerial Imagery

**DOI:** 10.3390/s25082534

**Published:** 2025-04-17

**Authors:** Guofeng Zhang, Yanfei Peng, Jincheng Li

**Affiliations:** School of Electronic and Information Engineering, Liaoning Technical University, Huludao 125105, China; 2203030329@stu.lntu.edu.cn (G.Z.); 472220574@stu.lntu.edu.cn (J.L.)

**Keywords:** unmanned aerial vehicle, small target detection, YOLOv8n

## Abstract

In unmanned aerial vehicle (UAV) aerial imagery scenarios, challenges such as small target size, compact distribution, and mutual occlusion often result in missed detections and false alarms. To address these challenges, this paper introduces YOLO-MARS, a small target recognition model that incorporates a multi-level attention residual mechanism. Firstly, an ERAC module is designed to enhance the ability to capture small targets by expanding the feature perception range, incorporating channel attention weight allocation strategies to strengthen the extraction capability for small targets and introducing a residual connection mechanism to improve gradient propagation stability. Secondly, a PD-ASPP structure is proposed, utilizing parallel paths for differentiated feature extraction and incorporating depthwise separable convolutions to reduce computational redundancy, thereby enabling the effective identification of targets at various scales under complex backgrounds. Thirdly, a multi-scale SGCS-FPN fusion architecture is proposed, adding a shallow feature guidance branch to establish cross-level semantic associations, thereby effectively addressing the issue of small target loss in deep networks. Finally, a dynamic WIoU evaluation function is implemented, constructing adaptive penalty terms based on the spatial distribution characteristics of predicted and ground-truth bounding boxes, thereby optimizing the boundary localization accuracy of densely packed small targets from the UAV viewpoint. Experiments conducted on the VisDrone2019 dataset demonstrate that the YOLO-MARS method achieves 40.9% and 23.4% in the mAP50 and mAP50:95 metrics, respectively, representing improvements of 8.1% and 4.3% in detection accuracy compared to the benchmark model YOLOv8n, thus demonstrating its advantages in UAV aerial target detection.

## 1. Introduction

In recent years, object detection technology has profoundly influenced computer vision, emerging as a vital and challenging subfield. The main goal of object detection is to precisely identify and locate instances of objects from predefined categories, with extensive applications in everyday life. Unmanned aerial vehicles (UAVs) have proven highly effective in domains such as security surveillance, precision agriculture, and civil infrastructure assessment [1]. In these fields, UAV-based object detection is crucial.

Object detection algorithms based on deep learning are generally divided into two categories: two-stage and single-stage approaches. Two-stage methods (e.g., R-CNN [2], Faster R-CNN [3], and Mask R-CNN [4]) first generate candidate regions, followed by classification and bounding box regression. While these approaches achieve high accuracy, they suffer from redundant computations, which can hinder inference efficiency. On the other hand, single-stage methods (e.g., YOLO [5,6], SSD [7], and RetinaNet [8]) directly extract image features for both classification and localization, offering a faster detection process while maintaining high accuracy. This makes single-stage algorithms particularly well-suited for real-world applications.

However, UAV aerial images often pose challenges, including complex backgrounds, target occlusion, insufficient lighting, and densely packed small targets, primarily resulting from factors such as long shooting distances and wide fields of view [9]. These challenges severely limit the accuracy of current detection algorithms.

To tackle the challenges mentioned earlier, Zhao et al. [10] proposed the Visibility-Enhanced DINO (VE-DINO) model, an extension of the DINO framework (Detection Transformer with Improved Denoising Anchor Boxes), which markedly enhances detection accuracy for heavily occluded objects in disaster scenarios. Zhu et al. [11] introduced a novel prediction head for YOLOv5 aimed at detecting targets across various scales. They replaced the traditional prediction head with a Transformer Prediction Head (TPH) to explore the potential of self-attention mechanisms in prediction. Liang et al. [12] proposed the Feature Fusion and Scaling-based Single Shot Detector (FS-SSD), which enhances the feature fusion module by adding a deconvolution branch and employing average pooling techniques to create a specialized feature pyramid, significantly boosting detection accuracy for small objects in UAV images. Guo et al. [13] introduced AugFPN, an innovative feature pyramid architecture that reduces the semantic gap between features of different scales prior to fusion using consistency supervision, while leveraging residual features to improve the extraction of invariant contextual information, thus minimizing information loss in the top feature maps of the pyramid. Fang et al. [14] developed the Cross-Modal Fusion Transformer (CFT), which incorporates attention mechanisms to extract image features using a transformer architecture, allowing the network to focus on global contextual information and enabling both intra-modal and inter-modal fusion. Liu et al. [15] presented the Scale and Location Sensitive (SLS) loss function, which adjusts the IoU loss weight based on the target scale to assist the detector in distinguishing targets of different sizes, while also incorporating a penalty term focused on the target’s center point to enhance localization accuracy. Yang et al. [16] proposed the Clustering Detection Network (ClusDet), which performs end-to-end detection through the Clustering Proposal Subnet-work (CPNet), Scale Estimation Subnetwork (ScaleNet), and a dedicated Detection Network (DetecNet), addressing the challenges of small target clustering and the uneven distribution of targets in UAV images. Du et al. [17] proposed a context-enhanced group normalization (CE-GN) layer, which replaces statistics derived from sparsely sampled features with global context statistics. Additionally, they designed an adaptive multi-layer masking strategy to optimize mask ratios across different scales, enabling compact foreground coverage and enhancing the detection accuracy and efficiency of small objects. Yang et al. [18] introduced a filtered progressive small object detection (FPSOD) model based on a progressive mechanism. This model leverages a soft-threshold filtering module guided by an attention mechanism to effectively remove redundant information from high-level feature maps while simultaneously enhancing the semantic representation of small objects. Ma et al. [19] developed the small object detection network (SESA-Net), incorporating an adaptive derivative mask (ADM) and a sample importance assessment (SIA) strategy. The ADM module suppresses the dominant response of large objects, redirecting focus to small object features, while the SIA strategy addresses the limitations of the IoU loss function by utilizing high-quality positive samples generated by the ADM module. Tian et al. [20] proposed a dual neural network review method that rapidly identifies missed targets in single-stage detection by classifying secondary features of suspected target regions, thereby achieving high-quality detection of small objects. However, under adverse weather conditions—particularly haze—the detection performance of existing models deteriorates significantly. To address this issue, Ren et al. [21] and Liu et al. [22] proposed distinct image dehazing algorithms. These novel techniques facilitate more effective target detection by UAVs under hazy atmospheric conditions.

This paper proposes an improved algorithm, YOLO-MARS, based on YOLOv8n. The main contributions of the proposed method are as follows:

1. Enhanced Residual Attention Convolution Module (ERAC): Compared to the traditional Conv module, the ERAC module enhances the ability to capture small target features by expanding the receptive field and introducing an attention mechanism. Additionally, the residual connection structure effectively mitigates the gradient vanishing problem, ensuring the stability of model training.
2. Parallel Depth-Aware Spatial Pyramid Pooling Module (PD-ASPP): Compared to the SPPF module, PD-ASPP employs a multi-branch parallel mechanism combined with depthwise separable convolution, reducing computational redundancy and enhancing the feature representation capability for multi-scale targets.
3. Shallow Guided Cross-Scale Feature Pyramid Network (SGCS-FPN): Addressing the shortcomings of existing feature pyramid networks that often lose small target information in deep networks, SGCS-FPN introduces shallow feature guidance branches to establish cross-scale semantic associations, significantly improving the detection performance for small targets.
4. Adjustable Weighted Intersection Over Union (WIoU): Compared to the traditional CIoU loss function, WIoU enhances the target localization regression mechanism by dynamically adjusting the positioning loss weight strategy. This improvement boosts the recognition accuracy and coordinate localization precision of the model in complex drone scenarios.

## 2. Related Works

**Figure 1:**
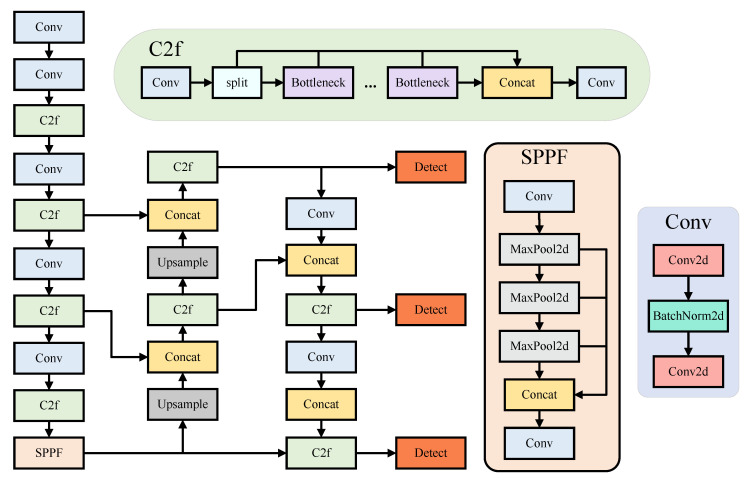
YOLOv8 network structure diagram.

YOLOv8 is the successor version of YOLOv5 released by Ultralytics on 10 January 2023, supporting a variety of computer vision tasks such as image classification and target detection. It supports a variety of computer vision tasks, such as image classification and object detection. The model is offered in five variants—YOLOv8n, YOLOv8s, YOLOv8m, YOLOv8l, and YOLOv8x—each distinguished by differences in network depth and width. As the number of parameters and computational requirements increase, detection accuracy improves, allowing the model to meet diverse task requirements and computational constraints. In light of the current application scenarios for drones, YOLOv8n exhibits superior portability owing to its streamlined network architecture, minimal computational resource requirements, and rapid execution speed. Consequently, the aim of this study is to enhance the YOLOv8n model. The YOLOv8n model’s network architecture comprises an input layer, a backbone network, a neck network, and a head network. Figure 1 illustrates the structural diagram of the YOLOv8 model.

The input component enriches the dataset using Mosaic data augmentation technology to enhance the model’s training effectiveness. Additionally, adaptive scaling techniques are applied to input images to improve the model’s robustness and generalization capabilities.

The backbone is primarily responsible for feature extraction and incorporates the CSP (Cross-Stage Partial) approach. It consists of the Conv module, C2f module, and SPPF module. The Conv module employs standard convolution combined with batch normalization and the SiLU activation function, effectively altering the resolution and channel count of the image to achieve superior feature extraction. The C2f module is inspired by the C3 module in YOLOv5 and the ELAN (Efficient Long-Range Attention Network) in YOLOv7. It utilizes enhanced skip-layer connections and additional split operations to enrich the gradient information within the model. The SPPF module performs feature fusion through pooling and convolution operations, adaptively integrating multi-scale feature information and enhancing the model’s representational capabilities.

The neck component is designed to fuse multi-scale feature information extracted by the backbone network. It combines a Path Aggregation Network (PAN) [23] and a Feature Pyramid Network (FPN) [24]. FPN strengthens semantic features via a top-down transmission mechanism, while PAN enhances localization information through a bottom-up transmission method. The integration of FPN and PAN achieves effective fusion of feature maps across different stages, significantly improving the model’s ability to detect objects at varying scales.

The head component transitions from anchor-based approaches to anchor-free methods, employing an anchor-free [25] decoupled head structure to separate classification and regression tasks, thereby improving model performance. For the loss function, BCE loss is utilized for classification loss, while CIoU is applied for bounding box regression loss. Additionally, a dynamic task-aligned assigner matching strategy is introduced to further enhance detection accuracy and generalization capabilities. This design enables the model to flexibly and efficiently handle diverse target scenarios.

## 3. Method

### 3.1. YOLO-MARS

**Figure 2:**
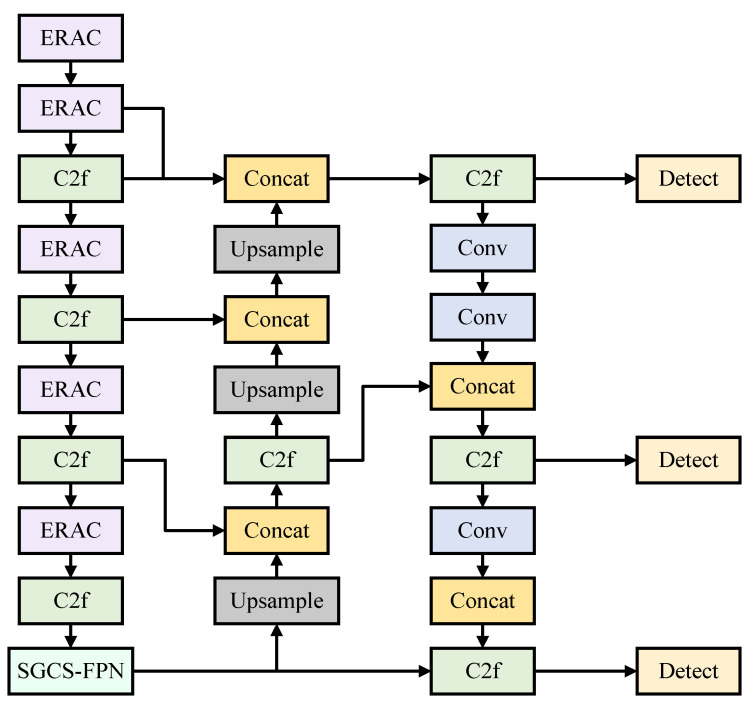
YOLO-MARS network structure diagram.

To address the challenge of low detection accuracy in aerial images, this study adopts YOLOv8n as the baseline model for improvement. First, the ERAC feature enhancement unit is introduced to expand the receptive field coverage. This unit integrates channel attention mechanisms to prioritize small target features and employs residual connections to enhance the stability of gradient propagation. Second, the PD-ASPP module is developed to enable multi-dimensional feature extraction via parallel paths, complemented by depthwise separable convolution to minimize redundant computations, thereby achieving precise recognition of multi-scale targets in complex scenes. Third, a multi-scale SGCS-FPN fusion framework is designed, incorporating shallow feature guidance branches and establishing cross-level semantic connections, which significantly mitigate the omission of small target information by deep networks. Finally, a dynamic WIoU evaluation mechanism is proposed, incorporating an adaptive penalty factor based on the spatial distribution characteristics of predicted boxes and ground truth boxes, thus enhancing the boundary localization stability for densely packed small targets in aerial imagery. The architecture of YOLO-MARS is illustrated in Figure 2.

#### 3.1.1. ERAC Module

**Figure 3:**
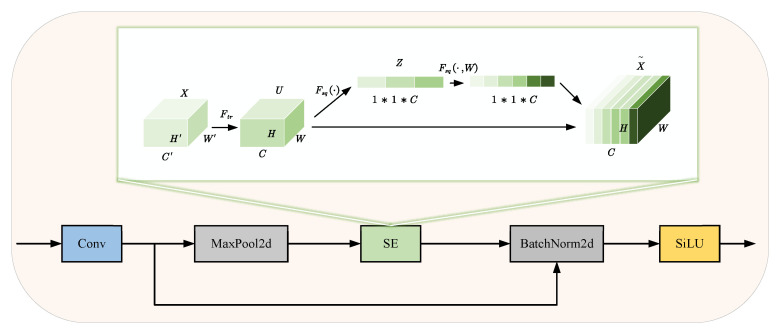
Schematic diagram of ERAC module.

Aerial images captured by unmanned aerial vehicles (UAVs) often contain a large number of densely distributed small targets within complex backgrounds. However, the Conv convolution modules in YOLOv8 exhibit limitations in effectively capturing small target features, which can result in significant feature loss. To mitigate this issue, this paper proposes the Enhanced Residual Attention Convolution (ERAC) module, the structure of which is illustrated in Figure 3.

In the ERAC module, MaxPool2d is first incorporated as an effective downsampling technique. By selecting the maximum value within the local regions of the feature map, MaxPool2d not only expands the network’s receptive field and reduces information redundancy but also enhances the ability to capture feature information from small targets.

Next, the SE attention module [26] is introduced. The SE attention mechanism learns adaptive channel weights and adjusts the contribution of each channel in the feature map based on task requirements. This enables the model to focus on informative channel features, thereby improving feature discrimination and enhancing model performance and accuracy. Additionally, the SE module effectively extracts global features, allowing features near the input layer to acquire a global receptive field. Furthermore, the SE attention mechanism introduces minimal additional parameters and computational overhead, demonstrating excellent flexibility and versatility.

Finally, a residual structure is integrated into ERAC. The residual structure mitigates the gradient vanishing problem, stabilizes the training of deeper neural networks, and preserves the information processed by the initial convolutional layers. This design facilitates the model’s ability to learn deep features more effectively.

Additionally, residual connections preserve information from the initial convolutional layers, allowing the model to acquire more representative deep-layer features while ensuring efficient gradient propagation.

#### 3.1.2. PD-ASPP Module

**Figure 4:**
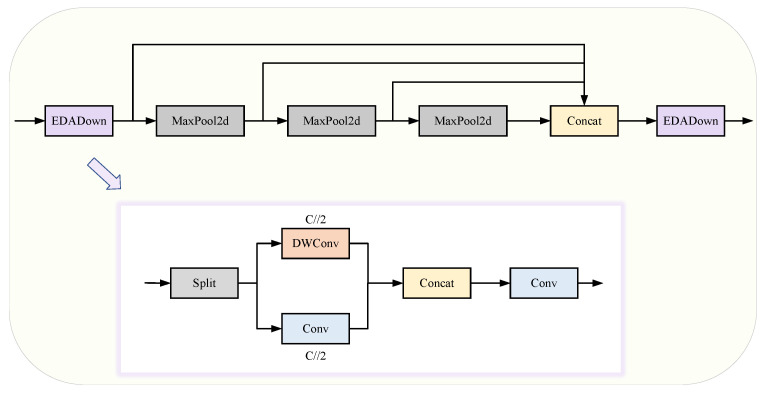
PD-ASPP module schematic diagram.

The significant scale variations of objects in aerial images and the high degree of similarity between small targets and backgrounds pose challenges for the SPPF module in effectively distinguishing them. In YOLO-MARS, inspired by ADown [27], we designed an efficient depth-aware downsampling module named EDADown to replace the original convolutional module in SPPF while also developing a lightweight feature enhancement module, PD-ASPP. The PD-ASPP module enhances the model’s feature representation capability by processing information pertaining to small targets through different path-ways. The specific structure of these modules is shown in Figure 4.

The ADown module utilizes pooling operations in place of traditional convolutions for downsampling. Through a branching architecture, the final feature maps preserve the original feature details while also integrating additional features processed through different paths. This design enhances the model’s ability to capture and express complex feature relationships.

In the EDADown module, building on the branched architecture of ADown, the original pooling operation is removed because the SPPF module already contains multiple pooling layers, thereby further simplifying the model structure. Additionally, one of the branches replaces the OBS module with a depthwise convolution (DWConv) module. This substitution not only preserves the feature fusion capabilities but also reduces the model’s parameter count. Depthwise convolutions perform convolution operations independently on each channel, capturing local spatial correlations within the input data. This enables the model to learn channel-specific features more effectively, ultimately improving its ability to capture spatial information across different dimensions.

#### 3.1.3. SGCS-FPN

In YOLOv8, shallow networks contain abundant information pertaining to small-target features. However, the low resolution and limited feature representation of small targets render them vulnerable to interference from medium and large objects during feature extraction. As the network depth increases, subtle shallow-layer features may be neglected, ultimately resulting in the loss of critical information pertaining to small targets in deep feature maps. This leads to frequent missed detections, thereby compromising detection performance relative to expectations. To address this issue and capture more shallow-layer features of small targets, we propose a scale fusion branch based on the PANet architecture. Specifically, a downsampling module and a Concat module are incorporated into the 14th layer of the neck section to extract feature scales from the first and second layers, thereby constructing a shallow-scale branch. Due to the introduction of an upsampling module, an additional downsampling module is subsequently introduced to ensure scale consistency across different levels. This configuration enables the acquisition of 160 × 160 feature maps to further extract the shallow-layer features of small targets. By integrating shallow-layer feature maps with deep-layer feature maps, the detection performance for small targets is significantly enhanced.

#### 3.1.4. WIoU

In YOLOv8, the bounding box regression loss function utilizes the CIoU (Complete Intersection over Union) loss function. Unlike the traditional IoU (Intersection over Union), CIoU not only considers the overlap between the predicted and ground-truth boxes but also incorporates the distance between their centers and the diagonal length of the smallest enclosing box. While the computation of CIoU resembles that of IoU, as both metrics evaluate overlap by dividing the intersection area by the union area, CIoU offers a more comprehensive assessment. The specific formula for calculating CIoU is presented in Equation (1):

(1) $$CIoU=IoU-\frac{\rho^{2}\left(A,B\right)}{c^{2}}-\alpha \nu$$

In the CIoU loss function, $\rho^{2}\left(A,B\right)$ represents the Euclidean distance between the centers of the predicted and ground truth boxes, *c* denotes the diagonal distance of the smallest enclosing box, *a* is the weighting function, and *v* stands for the penalty term.

The CIoU loss incorporates the aspect ratio of the predicted box and the ground-truth box rather than merely their width and height. When the aspect ratios of both boxes are identical, the CIoU value remains consistent. However, in real-world object detection scenarios, particularly for small-target detection, object scales exhibit significant variation. Factors such as lighting conditions, occlusions, and changes in viewpoint can cause significant alterations in the size, shape, and appearance of targets. In such cases, CIoU may fail to adequately address these variations, resulting in diminished detection performance.

To overcome the limitations of traditional loss functions, we introducethe Weighted Intersection over Union (WIoU) loss function [28] for bounding box regression. Initially, a distance attention mechanism is introduced based on the distance metric between anchor boxes and target boxes, resulting in WIoUv1, which integrates attention mechanisms. The calculation formulas for WIoUv1 are provided in Equations (2)–(4).

(2) $$\begin{array}{c} \begin{array}{ccc} \; & \; & L_{WIoU_{V1}}=R_{WIoU}L_{IoU} \end{array} \end{array}$$

(3) $$\begin{array}{c} \begin{array}{ccc} \; & \; & R_{WIoU}=\exp\left(\frac{{\left(x-x_{gt}\right)}^{2}+{\left(y-y_{gt}\right)}^{2}}{{\left(W_{g}^{2}+H_{g}^{2}\right)}^{*}}\right) \end{array} \end{array}$$

(4) $$\begin{array}{c} \begin{array}{ccc} \; & \; & L_{IoU}=1-IoU \end{array} \end{array}$$

where *x*, *y* is the center coordinate of the predicted box; $x_{gt}$, $y_{gt}$ is the center coordinate of the real box; $W_{g}$ and $H_{g}$ are the width and height of the real box; and Iou is the intersection and union ratio of the predicted box and the real box.

WIoUv2 introduces the monotonic focusing coefficient $L_{IoU}^{*}$, which effectively mitigates the influence of simple examples on the overall loss. However, during model training, as $L_{IoU}$ decreases, the gradient gain $L_{IoU}^{*}$ gradually diminishes, resulting in slower convergence. To address this issue and sustain a high convergence rate in the later stages of training, a moving average $\bar{L_{IoU}}$ is introduced as a normalization factor. The mathematical formulation of WIoUv2 is provided in Equation (5).

(5) $$\begin{array}{cc} \; & L_{WIoUv2}={\left(\frac{L_{IoU}^{*}}{\bar{L_{IoU}}}\right)}^{\gamma }\times L_{WIoUv1},\;\gamma >0 \end{array}$$

WIoUv3 introduces an outlier factor β to assess the quality of anchor boxes and leverages this factor to construct a non-monotonic focusing weight *r*. A smaller outlier factor β indicates higher-quality anchor boxes, assigning them smaller *r* values to reduce their weight in the loss function. Conversely, a larger β corresponds to lower-quality anchor boxes, assigning them smaller gradient gains to mitigate the adverse effects of low-quality anchor boxes. The mathematical formulation of WIoUv3 is provided in Equation (6). The definitions of β and *r* are provided in Equations (7) and (8).

(6) $$\begin{array}{cccc} \; & \; & L_{WIoU_{\nu 3}}=rL_{WIoU_{\nu 1}} & \; \end{array}$$

(7) $$\begin{array}{c} \begin{array}{ccc} \; & \; & r=\frac{\beta }{\delta \alpha^{\beta -\delta }} \end{array} \end{array}$$

(8) $$\begin{array}{c} \begin{array}{c} \begin{array}{ccc} \; & \; & \beta =\frac{L_{IoU}^{*}}{\bar{L_{IoU}}} \end{array} \end{array} \end{array}$$

where * means separating $W_{g}$ and $H_{g}$ from the calculation; α and δ are hyperparameters.

As a loss function, WIoUv3 employs a dynamic, non-monotonic mechanism to evaluate anchor box quality, enabling the model to focus more effectively on anchor boxes of moderate quality and enhancing its target localization capabilities. WIoUv3 addresses the inherent biases of traditional IoU-based losses, particularly in detecting small or highly variable objects.

## 4. Experiments and Analysis

### 4.1. Dataset

The VisDrone2019 dataset, captured by the AISKYEYE team from Tianjin University, covers a wide variety of environmental and weather conditions across 14 cities in China [29]. The dataset comprises a total of 10,209 images, including 6471 images in the training set, 548 images in the validation set, and 3190 images in the test set. It encompasses 10 target categories, including pedestrians, vehicles (such as bicycles, cars, vans, trucks, buses, and motorcycles), and other transportation forms (e.g., tricycles and shaded tricycles). The images vary in size from 480 × 360 pixels to 2000 × 1500 pixels, with small targets accounting for approximately 60% of the dataset. This characteristic makes it particularly well-suited for small target detection tasks in aerial imagery, as explored in this study. Additionally, the dataset contains a large number of small targets and substantial occlusion problems, which complicate detection, making it a challenging benchmark for model evaluation.

### 4.2. Experimental Environment and Training Parameters

To ensure consistency and enhance the credibility of the experimental results, all experiments conducted in this study employ the same experimental environment configuration and parameter settings. The specific experimental environment configuration is presented in Table 1.

Training the YOLOv8 network model involves several hyperparameters. The proper selection of these hyperparameters can effectively improve the model’s convergence speed and enhance its robustness. The parameters used in this paper are listed in Table 2.

### 4.3. Evaluation Metrics

To effectively evaluate the performance of the improved model, this study employs accuracy, recall, and mean average precision (mAP) as metrics to represent detection accuracy, with the parameter count used as a reference indicator.

Precision measures the proportion of samples correctly classified as positive when the model predicts a sample to belong to the positive class, as defined in Equation (9). Recall quantifies the proportion of positive samples correctly identified by the model relative to the total number of positive samples, as defined in Equation (10).

(9) $$Precision=\frac{TP}{TP+FP}$$

(10) $$Recall=\frac{TP}{TP+FN}$$

where TP denotes the number of correctly predicted targets belonging to the correct class, FP represents the number of incorrectly predicted targets classified as the wrong class, and FN refers to the number of incorrectly predicted targets belonging to the correct class.

The curve formed by recall and precision, with recall on the horizontal axis and accuracy on the vertical axis, is referred to as the P–R curve. Mean average precision (mAP) represents the area enclosed by the P–R curve and the coordinate axes, serving as an evaluation metric for assessing the model’s performance on the VisDrone2019 dataset. The calculation formula for mAP is provided in Equation (11).

(11) $$mAP=\frac{1}{n}\sum_{i=1}^{n}\int_{0}^{1}P\left(R\right)dR$$

where *n* represents the total number of categories.

mAP50 refers to the mean average precision (mAP) calculated at an intersection over union (IoU) threshold of 0.5. It evaluates a model’s detection performance using a relatively lenient criterion for bounding box matching. mAP50:95 by contrast, denotes the mean average precision averaged over multiple IoU thresholds ranging from 0.5 to 0.95 with increments of 0.05. This metric provides a more comprehensive evaluation of the model’s performance under increasingly stringent matching conditions.

### 4.4. Ablation Experiments

Ablation tests were performed on the VisDrone2019 validation set to rigorously evaluate the efficacy of the suggested enhancements. This subsection presents a comprehensive study of the effects of each upgrade on the overall efficacy of the proposed algorithm. The findings of the ablation experiment are displayed in Table 3. A refers to the baseline model YOLOv8n, B represents the integration of the ERAC module into the baseline model, C denotes the incorporation of the PD-ASPP module based on model B, D indicates the addition of the SGCS-FPN module to model C, and E represents the introduction of WIoUv3 into model D.

Analyzing the experimental results in Table 3 shows that, compared to the baseline model A, model B uses the ERAC module designed in this study, leading to increases in mAP50 and mAP50:95 of 1.8% and 0.9%, respectively, with a minimal increase in parameters. This indicates that the ERAC module performs well in enhancing the network’s receptive field, improving feature discrimination, and facilitating better gradient propagation, effectively capturing fine-grained features of small targets and significantly improving the detection accuracy in small target detection tasks. Based on model B, model C incorporates the PD-ASPP module designed in this study, which improves mAP50 and mAP50:95 by 0.9% and 0.2%, respectively, while reducing the number of parameters by 4.3%. This indicates that the PD-ASPP module enhances the model’s ability to recognize small targets and its representational capacity for targets of different scales through lightweight design, feature enhancement, and deep convolution optimization. Model D introduces the new feature pyramid network SGCS-FPN based on model C. Compared to model C, model D exhibits a significant performance improvement, with an increase of 5.4% in mAP50 and 3.3% in mAP50:95. This improvement is primarily due to SGCS-FPN’s ability to enhance the extraction of shallow small target features by introducing a scale fusion branch. Finally, model E utilizes WIoUv3 as the bounding box regression function, introducing weight factors and a dynamic non-monotonic mechanism, allowing the model to more precisely assess the quality of bounding boxes and overlap evaluation, further improving detection accuracy.

Overall, each improvement method yielded notable results, demonstrating that the improved YOLO-MARS algorithm substantially enhances the target recognition and detection capabilities from the UAV perspective.

### 4.5. Experimental Results Analysis

**Figure 5:**
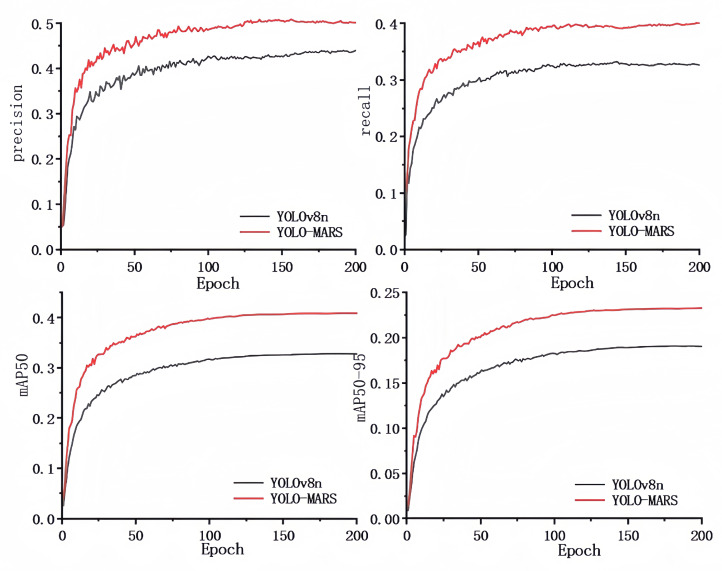
Performance indicators of YOLOv8n and YOLO-MARS training process.

To visually demonstrate the effect of the algorithm optimization, this paper presents a comparative analysis of YOLOv8n and YOLO-MARS during training on the VisDrone2019 dataset, focusing on key performance metrics such as precision, recall, mAP50, and mAP50-95. The results, as shown in Figure 5, indicate that YOLO-MARS outperforms the baseline model across all evaluation metrics, validating its superior performance in the target detection task.

**Figure 6:**
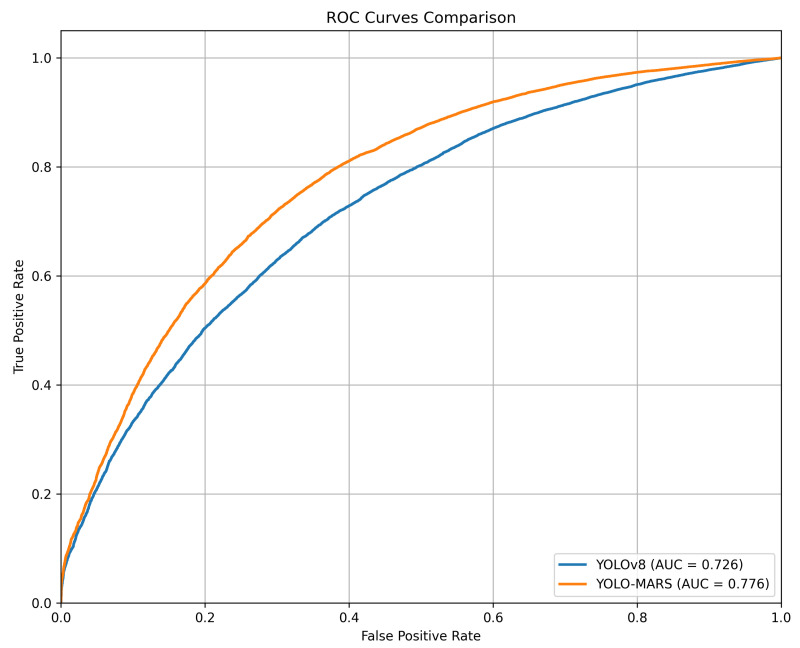
Comparison of YOLOv8n and YOLO-MARS ROC curves.

The ROC curve is a crucial tool for evaluating the performance of classification algorithms. It is generated by plotting the true positive rate (TPR) against the false positive rate (FPR) at various threshold settings. The area under the curve (AUC) serves as a quantitative measure of the ROC curve, with higher AUC values indicating better overall performance. Figure 6 presents a comparison of the ROC curves for YOLOv8n and the proposed YOLO-MARS. As shown, YOLO-MARS achieves an AUC of 0.776, outperforming YOLOv8n in detection capability.

**Figure 7:**
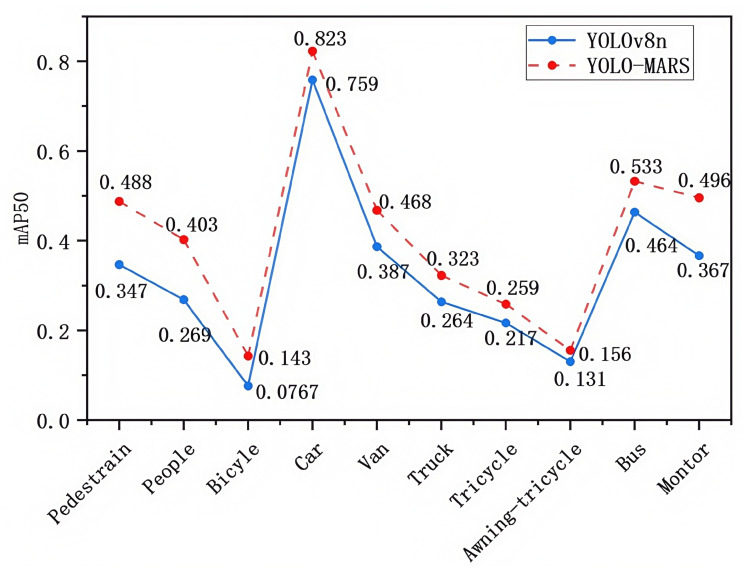
Comparison of average precision of different categories.

Figure 7 presents a comparative overview of the average detection accuracy of YOLOv8n and YOLO-MARS across ten target categories in the dataset. Specifically, for small target categories, YOLO-MARS demonstrated improvements in detection accuracy of 14.1%, 13.4%, 6.6%, 4.2%, and 2.5% for pedestrians, humans, bicycles, tricycles, and canopy tricycles, respectively, compared to YOLOv8n. In the medium target categories, the detection accuracy for cars, vans, and motorcycles increased by 6.4%, 8.1%, and 12.9%, respectively. For large target categories, the detection accuracy for trucks and buses improved by 5.9% and 6.9%, respectively. These experimental results indicate that YOLO-MARS not only enhances the detection accuracy of small targets but also significantly improves the detection performance of medium and large targets, indicating a well-rounded enhancement across target sizes.

### 4.6. Comparative Experiments

To further analyze the superiority of the improved algorithm, under the premise of ensuring the same dataset and experimental environment, the improved algorithm model YOLO-MARS was compared and verified with the current mainstream detection algorithms. The comparison algorithms include the YOLO series and its improved algorithms proposed in recent years, the QueryDet algorithm for small target detection, and the classic SSD, Faster-RCNN, etc. The experiment was carried out based on the VisDrone2019 dataset, and the results are shown in Table 4.

The experimental results presented in Table 4 show that, compared to classic detection algorithms such as SSD and Faster-RCNN, the proposed YOLO-MARS algorithm demonstrates significant advantages across various network precision metrics, with an mAP50 improvement of 17% and 7.7%, respectively. Compared to the QueryDet algorithm, which is specifically designed for small target detection, the YOLO-MARS algorithm not only has fewer parameters but also exhibits higher detection accuracy, with a 9.3% improvement in mAP50, making it more suitable for high-precision UAV detection scenarios. YOLOv5s has an mAP50 that is 7.6 percentage points lower than that of the proposed algorithm, while its parameter count is significantly higher. YOLOv7tiny’s mAP50 is 5.9 percentage points lower than that of the proposed algorithm, and its parameter count exceeds the proposed algorithm by 3.09 M. While YOLOv8s has 3.8 times the number of parameters of YOLO-MARS, its mAP50 is still 1.9% lower than that of the proposed algorithm. Compared with the YOLOv11n, YOLO-MARS increases the number of parameters by only 0.35 M, yet achieves substantial improvements across multiple performance metrics, including a 7.5% gain in the mAP50 score. Although the parameter count of YOLO-MARS is slightly higher than that of LUDY-N, it still outperforms LUDY-N by 4.7% in terms of mAP50. Furthermore, compared to the RFAG-YOLO, YOLO-MARS demonstrates superior detection performance, achieving a 2% improvement in mAP50 while reducing the number of parameters by 3.01 M.

The comparative experimental results demonstrate that the YOLO-MARS algorithm achieves higher detection accuracy, outperforming various mainstream detection algorithms and providing better results in target detection from the UAV perspective.

### 4.7. Visualization Results Analysis

**Figure 8:**
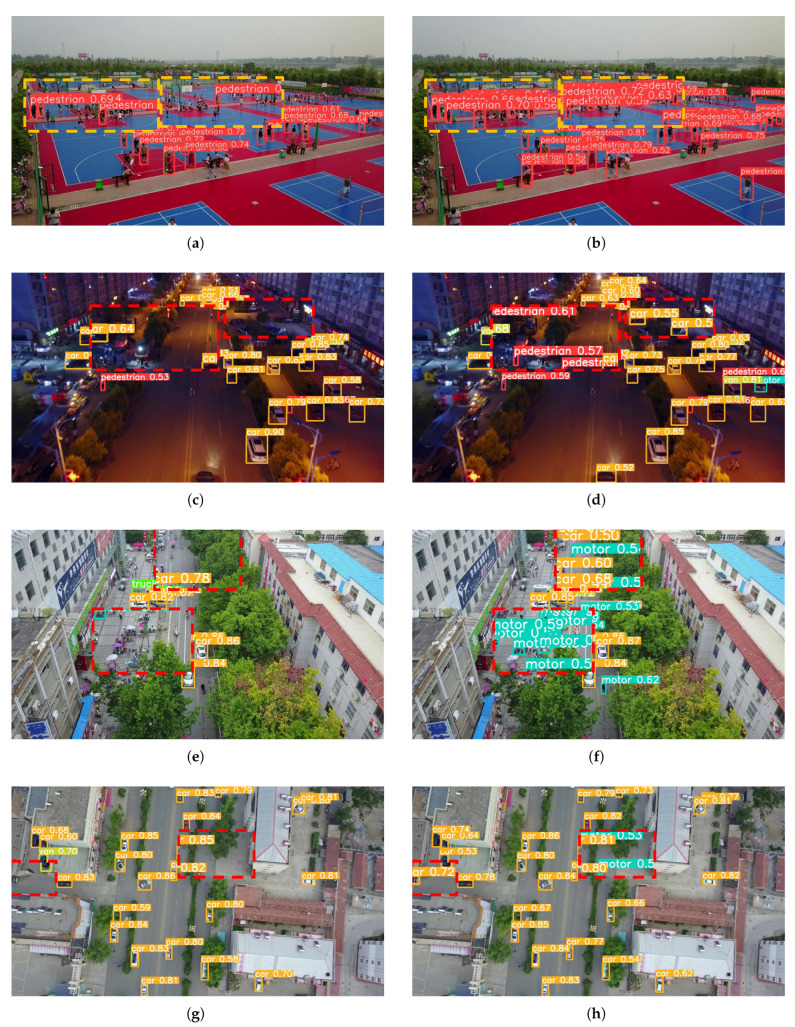
Comparison of object detection results on the VisDrone2019 dataset. (**a**,**c**,**e**,**g**) Detection results obtained by YOLOv8n. (**b**,**d**,**f**,**h**) Detection results obtained by our proposed YOLO-MARS model.

To evaluate the detection performance of the proposed algorithm in practical applications, representative scenes from the VisDrone2019 dataset were carefully selected for visual demonstration. Figure 8 com-pares the detection performance of YOLOv8n and the proposed algorithm YOLO-MARS across four scenarios: target-dense, nighttime, occlusion, and high-altitude, with each scenario illustrated by paired sub-figures for both models.

In the high-density target scenario, as shown in Figure 8a,b, the crowd is highly dense with significant mutual occlusion, and small tar-gets frequently overlap. Figure 8a presents the detection results of YOLOv8n, revealing numerous false positives and missed detections attributable to scene complexity. In contrast, Figure 8b demonstrates that YOLO-MARS significantly reduces these errors and successfully detects objects missed by YOLOv8n, highlighting its improved robustness in dense environments.

For nighttime detection, Figure 8c,d illustrate the performance under limited lighting conditions, in which target features become blurred, rendering details difficult to discern. Conversely, Figure 8d reveals that YOLO-MARS maintains accurate classification, thereby outperforming the baseline model through effective adaptation to low-light conditions.

In occlusion scenarios, depicted in Figure 8e,f, targets are frequently obscured by trees or other objects, resulting in indistinct boundaries and loss of critical features. Figure 8e indicates that YOLOv8n experiences varying degrees of missed detections, resulting in unsatisfactory performance. Conversely, Figure 8f shows that YOLO-MARS more accurately identifies occluded vehicles, motorcycles, and pedestrians, demonstrating superior detection capabilities in handling occlusions.

In high-altitude shooting scenarios, where the number of pixels representing targets decreases, features become less distinct, and background information is more prominent, Figure 8g,h provide a comparative analysis. Figure 8g illustrates that the baseline model YOLOv8n continues to experience false positives and missed detections. In contrast, Figure 8h highlights that YOLO-MARS accurately recognizes specific target categories, showcasing its enhanced performance in challenging high-altitude conditions.

**Figure 9:**
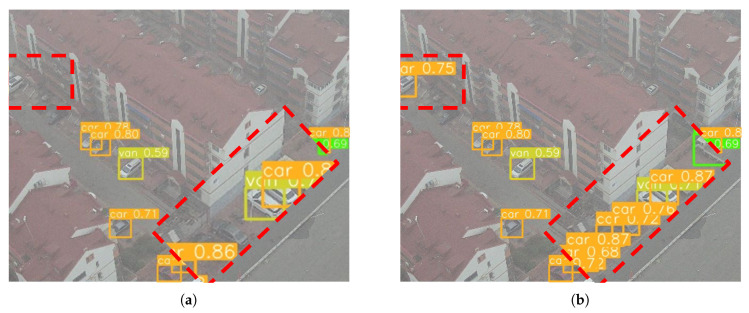
Comparison of target detection results in hazy weather. (**a**) Detection results obtained by YOLOv8n. (**b**) Detection results obtained by our proposed YOLO-MARS model.

Figure 9 illustrates the detection results of YOLOv8n and YOLO-MARS under haze weather conditions. The presence of haze causes targets to appear blurred and challenging to identify. In the detection results of YOLOv8n, a number of targets are missed. In contrast, YOLO-MARS exhibits superior performance, successfully detecting mutually occluded and overlapping objects in most scenarios. This demonstrates its robustness and reliability under haze weather conditions.

### 4.8. Extended Experiments

To comprehensively evaluate the generalization capability of the improved model, this paper further tested its performance on the HIT-UAV dataset [32]. The HIT-UAV dataset, specifically developed for high-altitude object recognition using drones, comprises a rich collection of infrared images. It is partitioned into a training set containing 2028 images, a validation set with 290 images, and a test set of 580 images. The experimental results are summarized in Table 5.

As demonstrated in Table 5, YOLO-MARS outperforms YOLOv8n across all metrics, achieving improvements of 7.7%, 5.1%, 4.9%, and 2.4% in precision, recall, mAP50, and mAP50:95, respectively. These enhancements indicate higher detection accuracy and a reduced missed detection rate. Furthermore, compared to SSD, Faster-RCNN, YOLOv5n,and YOLOv6n, YOLO-MARS exhibits a notable improvement in mAP50 by 13.1%, 15%, 6.7%, and 6.4%, respectively.

**Figure 10:**
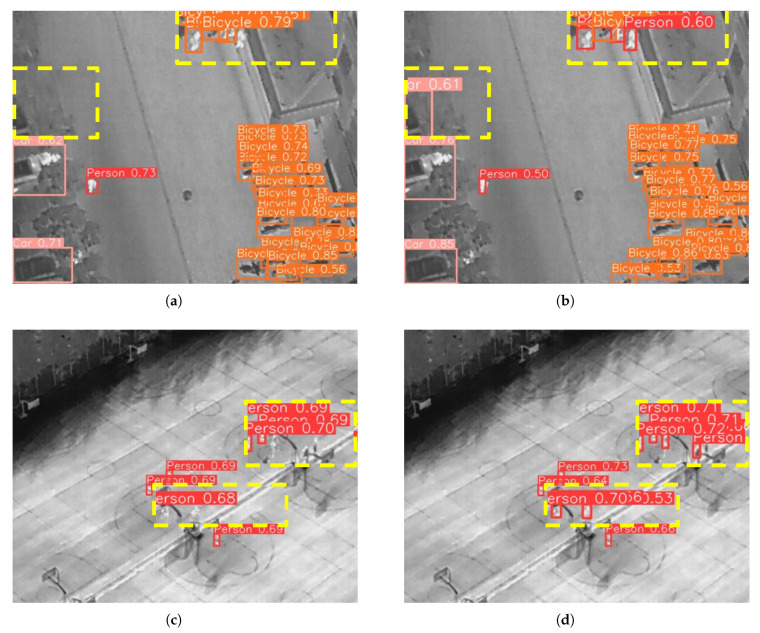
Comparison of object detection results on thee HIT-UAV dataset. (**a**,**c**) Detection results obtained by YOLOv8n. (**b**,**d**) Detection results obtained by our proposed YOLO-MARS model.

Figure 10 illustrates the detection performance of YOLOv8n and YOLO-MARS on the HIT-UAV dataset. Specifically, Figure 10a,c showcase the detection results of YOLOv8n, while Figure 10b,d showcase the results of YOLO-MARS. It is evident that YOLOv8n struggles with missed detections in infrared images, particularly for densely packed and occluded targets. In contrast, YOLO-MARS successfully identifies these targets.

In conclusion, the proposed YOLO-MARS model exhibits significant advantages in detecting small targets, achieving a lower missed detection rate, robust generalization ability, and broad applicability for small target detection tasks.

## 5. Conclusions

To tackle the challenge of low detection accuracy for small targets in UAV imagery, an improved YOLO-MARS based on YOLOv8n is proposed in this study. It effectively ad-dresses issues such as small target sizes, insufficient feature information, and low detection accuracy caused by dense distribution and occlusion in UAV imagery, resulting in a significant improvement in target detection performance. First, the ERAC module is designed to increase the network’s receptive field, enhance feature discrimination, and improve training stability. Next, the PD-ASPP module is introduced, which integrates multi-scale features to reduce the number of parameters while enhancing local spatial modeling ability and improving small target recognition. Then, the SGCS-FPN structure is pro-posed, incorporating a scale fusion branch to effectively enhance the utilization of shallow features, thus boosting small target detection capabilities. Finally, the WIoU loss function is introduced, improving the accuracy of boundary box regression through weighted coefficients and a dynamic non-monotonic mechanism.

In future research, model optimization techniques such as model pruning and knowledge distillation will be investigated further to reduce computational resource consumption while ensuring stable detection accuracy. Additionally, the introduction of super-resolution techniques will be considered to enhance small target detection performance by improving the quality of input images.

## Figures and Tables

**Table 1:** Experimental Environment Configuration.

Parameters	Configuration
Operating systems	Linux
CPU	AMD EPYC 9754 (Amd, CA, USA)
GPU	NVIDIA RTX 3090 (Nvidia, CA, USA)
Deep learning architecture	Pytorch 1.10.0 + Cuda 11.2

**Table 2:** Training Parameter Settings.

Parameters	Setup
Image size	640 × 640
Epochs	200
Learning rate	0.01
Momentum	0.937
Weight decay	0.0005
Batch size	16

**Table 3:** Results of ablation experiments on VisDrone2019 dataset.

Model	Params (M)	Precision (%)	Recall (%)	mAP50 (%)	mAP50:95 (%)
A	3.01	43.6	32.9	32.8	19.1
B	3.02	46.3	34.2	34.5	20.0
C	2.89	46.6	35.9	35.4	20.2
D	2.93	49.6	39.8	40.8	23.5
E	2.93	50.2	40.0	40.9	23.4

**Table 4:** Comparative experiments on the VisDrone2019 dataset.

Model	Params (M)	Precision (%)	Recall (%)	mAP50 (%)	mAP50:95 (%)
SSD	24.4	21.0	35.5	23.9	10.2
Faster-RCNN	41.2	45.5	33.8	33.2	17.0
QueryDet	18.9	41.4	33.4	31.6	17.4
YOLOv5s	7.2	44.8	34.1	33.3	33.2
YOLOv7-tiny	6.02	47.5	36.2	35.3	19.6
YOLOv8n	3.01	43.6	32.9	32.8	19.1
YOLOv8s	11.12	50.4	37.1	39.1	23.6
YOLOv11n	2.58	43.3	32.3	32.1	18.7
LUDY-N [30]	2.81	47.0	34.7	35.2	-
RFAG-YOLO [31]	5.94	49.6	37.8	38.9	23.1
YOLO-MARS	2.93	50.2	40.0	40.9	23.4

**Table 5:** Detection performance comparison on HIT-UAV dataset.

Model	Precision (%)	Recall (%)	mAP50 (%)	mAP50:95 (%)
SSD	75.1	67.8	72.1	41.89
Faster-RCNN	73.7	67.5	70.2	40.2
YOLOv5n	83.4	72.1	78.5	51.5
YOLOv6n	85.6	71.6	78.8	51.3
YOLOv8n	83.0	73.7	80.3	53.0
YOLO-MARS	90.7	78.8	85.2	55.4

## Data Availability

The data presented in this study are available upon request from the corresponding author.
